# Conduct Problems and Hair Cortisol Concentrations Decrease in School-Aged Children after VIPP-SD: A Randomized Controlled Trial in Two Twin Cohorts

**DOI:** 10.3390/ijerph192215026

**Published:** 2022-11-15

**Authors:** Jana Runze, Irene Pappa, Marinus H. Van IJzendoorn, Marian J. Bakermans-Kranenburg

**Affiliations:** 1 Clinical Child & Family Studies, Faculty of Behavioral and Movement Sciences, Vrije Universiteit, 1081 HV Amsterdam, The Netherlands; 2 Leiden Consortium on Individual Development, Leiden University and VU Amsterdam, 2311 EZ Leiden, The Netherlands; 3 Department of Psychology, Education, and Child Studies, Erasmus University Rotterdam, 3062 PA Rotterdam, The Netherlands; 4 Research Department of Clinical, Education and Health Psychology, Faculty of Brain Sciences, UCL, London WC1E 6BT, UK; 5 Institute of Psychological, Social and Life Sciences, ISPA Lisbon, 1149-041 Lisbon, Portugal; 6 Center for Attachment Research, The New School for Social Research, New York, NY 10011, USA

**Keywords:** VIPP-SD, hair cortisol concentrations (HCC), stress, HPA-axis, externalizing behavior, children, RCT, neuroendocrine system

## Abstract

The Video-feedback Intervention to promote Positive Parenting and Sensitive Discipline (VIPP-SD) is effective in increasing parental sensitivity and sensitive discipline, and aims to decrease child behavior problems. Changes in quality of parenting may be accompanied by effects on child stress levels. However, studies of VIPP-SD effects on child behavior problems have shown mixed results and there are no studies to date of the effect of the intervention on children’s stress levels, as measured by hair cortisol concentration (HCC). Furthermore, differences in intervention effectiveness may be explained by differential susceptibility factors. We hypothesized that the effects of the VIPP-SD on child behavior problems might be moderated by currently available child polygenic scores of differential susceptibility (PGS-DS). In the current pre-registered trial, we randomly assigned 40% of *n* = 445 families with school-aged twin children to the intervention group. The VIPP-SD was successful in decreasing both children’s conduct problems and HCC. Effects were not moderated by available child PGS-DS. We conclude that a brief, home-based video-feedback parenting intervention can decrease child behavior problems and affect the child’s stress-related neuroendocrine system as assessed with hair cortisol. In future studies, more specific PGS-DS for externalizing behaviors should be used as well as parental PGS-DS.

## 1. Introduction

Meta-analytic evidence has shown that the Video-feedback Intervention to promote Positive Parenting and Sensitive Discipline (VIPP-SD) is effective in increasing parental sensitivity, parental sensitive discipline, and child attachment security [1,2]. Parenting and attachment are important factors in the development and continuity of children’s stress regulation and externalizing behavior problems [3,4,5]. High-quality parenting and secure attachments have been found to predict lower levels of externalizing behaviors [6] and to buffer the stress reactivity of the hypothalamic–pituitary–adrenocortical (HPA) axis [4,7,8]. In the current randomized controlled trial (RCT) in two cohorts of families with twins, we therefore examined the effects of the VIPP-SD on children’s conduct problems and hair cortisol concentrations (HCC).

During the VIPP-SD, parents learn tools such as distraction and inductive discipline, in order to respond adequately to difficult child behavior, which helps decreasing problem behavior in the long term [1]. Moreover, parents learn to see even subtle signals of proximity-seeking behavior in their child and to react promptly and adequately, which should help decreasing child stress levels [1]. Although the effects of the VIPP-SD on parental sensitivity and secure attachment have been supported in several randomized trials and confirmed in a meta-analysis of 25 randomized controlled VIPP-SD studies, the evidence for the effects of VIPP-SD on children’s externalizing behavior remains inconclusive [2]. The most recent and largest pragmatic randomized trial conducted within the context of the British National Health Service (NHS) including more than 300 families with toddlers at risk for externalizing problems showed a significant effect of VIPP-SD on conduct problems [9]. Similarly, some previous research showed the effects of parenting interventions on children’s stress regulation but meta-analytic results were equivocal. The Attachment and Biobehavioral Catch-up (ABC) intervention with 4- to 6-year-old children involved with Child Protective Services (CPS) resulted in more typical salivary diurnal cortisol levels in the intervention group [10]. Also, in 1- to 3-year-old children with high externalizing behavior, diurnal cortisol production as measured in saliva samples decreased after the VIPP-SD, specifically in carriers of the DRD4 7-allele [11]. However, in a recent meta-analysis of 19 parenting intervention studies, no overall significant effect of parenting interventions on child salivary cortisol levels was found [12]. Further work is needed to provide more robust evidence for the effectiveness of attachment-based parenting interventions on child externalizing behavior and stress regulation.

An avenue not yet explored in parenting intervention studies on hormonal stress regulation is the use of hair cortisol concentrations (HCC, [13]). In developmental research, cortisol has mostly been measured using saliva, but salivary cortisol only provides a snapshot of cortisol levels on a particular day [14]. However, cortisol levels may vary strongly from day to day as they are influenced by many factors, such as food intake and activity [15]. Furthermore, only chronic stress indicated by long-term deviations in cortisol levels as assessed with HCC across several months may be problematic [16]. To our knowledge, only one small RCT including 25 parents and their children investigated the effect of compassion training in parents on children’s HCC and found decreased HCC in the intervention group [17].

Another topic to explore more thoroughly in parenting interventions is the moderating role of children’s genetic differential susceptibility to parenting [18,19]. Highly susceptible children are supposed to show more psychological problems when exposed to adverse environments, but also to benefit more from (experimentally) enhanced caregiving environments [20]. A promising approach to genetic differential susceptibility is the use of polygenic scores (PGSs) derived from genome-wide association studies (GWAS) to compute markers of differential susceptibility [21,22]. Keers and colleagues [22] were the first to develop a genome-wide association-based PGS for differential susceptibility to anxiogenic environments. Based on within-pair variability in emotional problems in monozygotic twins, they identified SNPs of environmental susceptibility and combined these in a PGS. This PGS was used to test the differential effects of treatment [22] and a Family Check-Up intervention on children’s internalizing symptoms [23]. In the absence of a specific PGS for susceptibility to externalizing-inducing environments, we decided to use the PGS for anxiogenic environments as a possible moderator of VIPP-SD effects as externalizing and internalizing problems usually show substantial correlations. In addition, for exploratory purposes, we computed a PGS based on a GWAS of susceptibility to environmental stress and adversity in adults (SESA) [24] that might also be a proxy for differential susceptibility to parenting.

In sum, we implemented the VIPP-SD intervention in a randomized controlled trial on two twin samples (Leiden Consortium on Individual Development (L-CID), total *n* = 890) measuring children’s conduct problems and HCC before and after the intervention. We expected that, after the intervention, children in the intervention group would exhibit fewer conduct problems compared to children in the control group (H1). Furthermore, we expected that, after the intervention, children’s HCC would be significantly lower in the intervention group as compared to the control group (H2). Lastly, we hypothesized that the intervention effects on HCC and conduct problems would be moderated by putative child polygenic scores for differential susceptibility (H3), with stronger intervention effects for children with higher polygenic scores.

## 2. Materials and Methods

### 2.1. Participants

**Figure 1:**
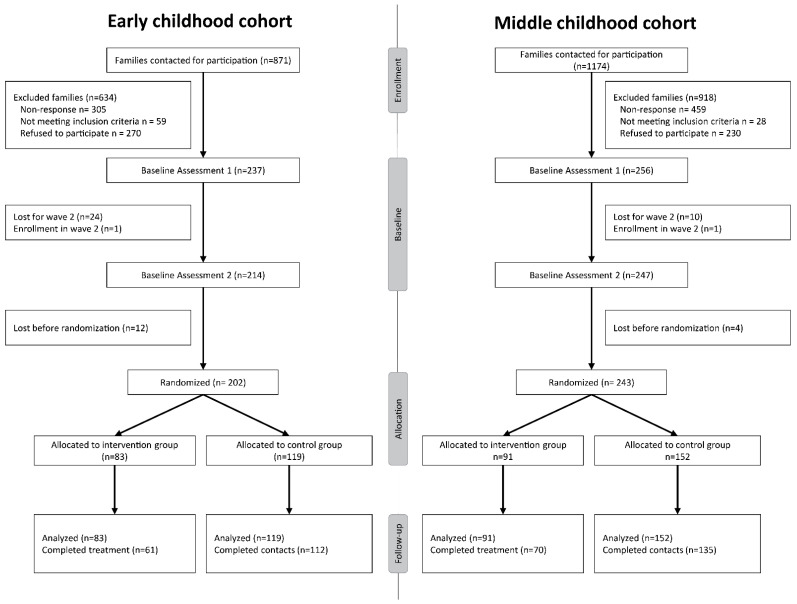
Flow chart of the randomized controlled trial with VIPP-SD in both twin cohorts. Number of participants reflects the number of families (including two twin children).

Participants were part of the L-CID project, a longitudinal intervention study with families with twins belonging to one of two cohorts: an early childhood cohort (*n* = 237 families) and a middle childhood cohort (*n* = 256 families) (Figure 1). At the first measurement, the children of the early childhood cohort were on average 3.76 years old (SD = 0.57) and the children of the middle childhood cohort were on average 7.92 years old (SD = 0.66). Sixty percent of the children were monozygotic twins and forty-five percent were male. Descriptive statistics for both cohorts and the full sample can be found in Table 1.

### 2.2. Procedure

Through municipality records, twin families from the western region of the Netherlands were contacted. Families were selected if the twins had the same sex, their parents were Dutch speaking, and parents as well as grandparents were born in Europe. Families with same sex twins were selected to prevent within-twin pair differences due to different sex, which would also lower the power of statistical analyses. The occurrence of congenital disability, psychological disorder, chronic illness, hereditary disease, visual/hearing impairment, or an IQ of <70 led to exclusion. Families received an invitation letter and an information brochure. Parents who indicated a willingness to participate were called to check the inclusion criteria and to provide more information about the study. For a detailed description of the recruitment, see Euser et al. [25] for the early childhood cohort and Van der Meulen et al. [26] for the middle childhood cohort. Six yearly visits were scheduled (alternating home and laboratory visits) with additional ambulatory assessments. The current study used data from the first, second, third, and fourth time of measurement (T1, T2, T3, T4) of the early childhood cohort (collected in 2014–2018) and middle childhood cohort (collected in 2015–2019). The intervention took place between T2 and T3. One month after the final intervention or control sessions had taken place, data for T3 were collected.

Ethical approval for the study was provided by the central committee on research involving human subjects (CCMO; Early childhood cohort NL49069.000.14, Middle childhood cohort NL50277.058.14). The study adheres to the CONSORT guidelines (see Appendix A). The trial, the study design, and the analysis were pre-registered (pre-registration).

### 2.3. Intervention

#### 2.3.1. Randomization

Using a computer-generated blocked randomization sequence, we randomized the sample at a ratio of 2:3 at the family level stratified by timing of the intervention and twin sex. A ratio of 2:3 was chosen as we had to restrict the number of families receiving the intervention due to limited resources. We randomized the sample after T2 to minimize selective attrition. The researcher who assigned the families to one of both conditions was not involved in data collection, coding or analysis. Interveners and families were blind to the condition before randomization but not afterwards due to the open label design. Coders and researchers involved in data coding or analysis were blind to the allocation. In total, 174 (39%) families were allocated to the intervention group and 271 (61%) families to the control group.

#### 2.3.2. VIPP-SD for Twins

We implemented the Video-feedback Intervention to promote Positive Parenting and Sensitive Discipline (VIPP-SD, adapted for twin families, [27]) between T2 and T3 in 39% of the families randomly assigned to the intervention group. The VIPP-SD is comprised of five biweekly sessions at the family’s home conducted by an intervener. In each session, the intervener first videotaped 15 minutes of parent–child interaction and then gave feedback on the video-recorded interaction of the previous session. During feedback, positive and successful interaction moments were highlighted, and alternatives for insensitive interactions were discussed with the parents. Each session had its own theme (see [1] for an overview of the themes). We invited the partner of the primary parent to the final session in order to support the primary parents’ implementation of positive parenting behaviors, according to protocol. As we conducted the intervention in twin families, minor adaptations to the original VIPP-SD program were made to address the challenges faced by the parents of twins. These adaptations included issues regarding dividing attention to both children at the same time, and dealing with jealousy and competition among the twin children. Also, toys and games were adapted to the situation of interacting with twins. For a detailed description of the intervention, see [27,28]. 

#### 2.3.3. Control Condition

Families in the control condition received five phone calls following a standard protocol parallel to the intervention sessions, to ensure the same number of contact occasions. Using a semi-structured interview, families were asked general and specific questions about their children’s development. When parents asked for advice on parenting issues, they were referred to online information or, in case of twin-specific questions, to the Dutch organization for parents with multiples (NVOM).

### 2.4. Measures

#### 2.4.1. Strengths and Difficulties Questionnaire (SDQ; [29])

Both parents completed the SDQ for both children, a 24-item questionnaire which consists of five subscales, one of which measures conduct problems (five items). Items are scored on a 3-point scale (“not true”, “somewhat true” or “certainly true”). An example item of the conduct problems scale is “often fights with other children or bullies them”. The reports of both parents correlated significantly (between r = 0.42 and r = 0.55); therefore, mean scores were computed. Cronbach’s alpha ranged between 0.65 and 0.82 across all time points and cohorts (see Table S1).

#### 2.4.2. Hair Cortisol Concentrations (HCC)

Hair samples were collected by a trained research assistant during the lab or home visit. Collecting hair is a non-invasive method, because only small amounts of hair are needed. Several months of cortisol secretion can be assessed [30]. As hair grows approximately 1 cm per month, every 1 cm segment of hair represents the past month. To collect hair samples, a strain of hair at the base of the vertex posterior of the scalp was selected and cut right at the scalp. Hair samples were put into foil and stored at a dark location at room-temperature until sent to the Dresden Lab Service GmbH in Germany for analysis. The most proximal 2 cm of hair were sectioned, representing cortisol production in the past two months. Liquid chromatography–mass spectrometry (LC–MS/MS) was used for cortisol quantification [31]. The lower limits of quantification were below 0.1 pg/mg. The inter- and intra-assay coefficients of variance were below 10%. For more details regarding the analysis, see [31].

#### 2.4.3. Genotyping and Imputation

Saliva samples were collected from the children at T2. The DNA was genotyped by the Genetic Laboratory of the Department of Internal Medicine (Population Genomics) at Erasmus MC using the GSA-MD array (version 3). The DNA QC was performed in PLINK [32]. After genotyping, the 1000 Genomes Project (phase III release version 5) was used to apply a two-step genotype imputation. The genetic ancestry of the children participating in this study was characterized using the genomic components equivalent to the principal components (PCs) of Europeans (CEU population). The first 5 PCs of the European-only sample were subsequently used as covariates in our analyses to adjust for spurious population stratification.

#### 2.4.4. Polygenic Score (PGS) of Differential Susceptibility

We computed two different polygenic differential susceptibility scores. We used the GWAS summary statistics of Keers et al. [22] to obtain a PGS of environmental susceptibility (PGS-ES). For secondary analyses, we used a PGS for the ‘susceptibility to environmental stress and adversity’ (SESA) cluster, as a potentially relevant construct of differential susceptibility (subsequently called PGS-SESA) [24]. For both PGSs we used the PRSice software. The GWAS summary statistics served as the base sample, and L-CID was the target sample. Only autosomal SNPs were used, since there is no consensus for the sex chromosomes [33]. The PGSs were calculated using clump r^2^ = 0.1, 250 kb at different *p*-value thresholds (i.e. 0.20, 0.10, 0.05, 0.01, and 0.001). We tested the PGSs using linear regression models and selected the *p*-value threshold of the PGSs that explained most variance based on the largest R^2^. The PGSs under the best *p*-value threshold were subsequently used for further analyses (see Table S2a,b) [33]. For the PGS-ES, the best *p*-value threshold for HCC was 0.05 and for conduct problems 0.2. Regarding the PGS-SESA, the best *p*-value threshold for HCC was 0.05 and for conduct problems was 0.001.

#### 2.4.5. Control Variables

Cortisol levels in hair can be influenced by several environmental factors, which we controlled for. These were sex, BMI, socio-economic status, the number of persons in a household, ethnicity, hair color, last hair wash, and frequency of hair washing [13,30]. Although covariate adjustment is not necessarily needed in randomized trials, we controlled for them as this can increase power [34]. In the early childhood cohort, children were approximately 5 years old at the time of the intervention, whereas in the middle childhood cohort they were approximately 10 years old at the time of the intervention, so we investigated whether age was a moderator of the intervention effects.

### 2.5. Data Analysis

We conducted intent-to-treat analyses and applied multilevel models with robust full maximum likelihood estimation using MPlus [35]. We had three levels (time of assessment, child, family). Each child had four data points for conduct problems and two data points for HCC (repeated measures), and the twin children were nested within families. We computed intraclass correlation coefficients (ICCs) to estimate the proportion of variance explained by the three levels. First, we computed the ICCs by fitting an intercept-only model with three levels and an unrestricted within-subject (co)variance structure separately for conduct problems and HCC. In the next step, we fitted growth models. For conduct problems, time and time^2^ were added as fixed and random effects. Condition, time^2^ × condition, time^2^ × condition × PGS-ES and time^2^ × condition × PGS-SESA were included as fixed effects. For HCC, time was added as fixed and random effect. Condition, time × condition, time × condition × PGS-ES and time × condition × PGS-SESA were included as fixed effects. We standardized all predictor variables before inclusion. We conducted the following sensitivity analyses: (1) including only those participants who received all five intervention sessions (81%), (2) including only families in which mothers are the primary caregivers (92%), and (3) for both cohorts separately.

We computed the a priori power using G*Power 3.1 [36] based on a repeated measures MANOVA with two outcome variables, an α = 0.05 and an effect size of f = 0.37, based on the effect size of Poehlmann-Tynan et al. [17]. With a sample size of 798 (both cohorts, sample size at T4), the a priori power was excellent (0.99) for detecting main effects. Even with a small effect size of f = 0.10, the power was good (0.81) for detecting main effects. Due to non-convergence issues, we adapted our original pre-registered analysis plans. Please see the Supplementary Materials for a detailed description of deviations from the preregistration.

## 3. Results

### 3.1. Preliminary Analyses

Means and standard deviation of the outcome variables can be found in Table 2. For hair cortisol, the distributions showed 19 outliers and for conduct problems 20 outliers (M+/− > 3.29 SD), which were winsorized, receiving a value between the highest/lowest non outlying value and the mean +/− 3.29 SD (Table S3). Of all the possible covariates (see Table S4a for descriptive statistics), BMI, the age of the child, the sex of the child, the frequency of washing the hair and the time of the last hair wash emerged as significant covariates for cortisol (Table S4b,c). The inclusion of these covariates, and the five PCs, led to the non-convergence of the model. Therefore, we conducted a regression analysis with the covariates as predictors and cortisol as outcome (R^2^ = 14.7%) and used the residuals as the outcome variable in subsequent analyses. There were missing data in both outcome variables (ranging from 2.2% to 17.6%, see Table S3). Little’s MCAR test was significant (X^2^ = 64.91, *p* < 0.001), indicating that data were not missing completely at random. The missing data were subsequently imputed using Expectation-Maximization single imputation. Data were imputed using the EM option in SPSS with a maximum of 25 iterations and intervention group, age, zygosity, and sex as predictors.

### 3.2. Main Results

For conduct problems, the ICC in the intercept-only model with three levels revealed that 11% of the variance in conduct problems could be attributed to the family level, meaning that conduct problems within a family were more similar to each other than conduct problems between families. Less than 1% percent could be attributed to the child level, meaning that conduct problems across time points within a child were similar compared to scores from the twin sibling. The same was true for HCC problems: the ICC in the intercept-only model with three levels revealed that 23% of the variance in HCC could be attributed to the family level and less than 1% could be attributed to the child level. In subsequent analyses, we therefore included only family and time as levels. 

**Figure 2:**
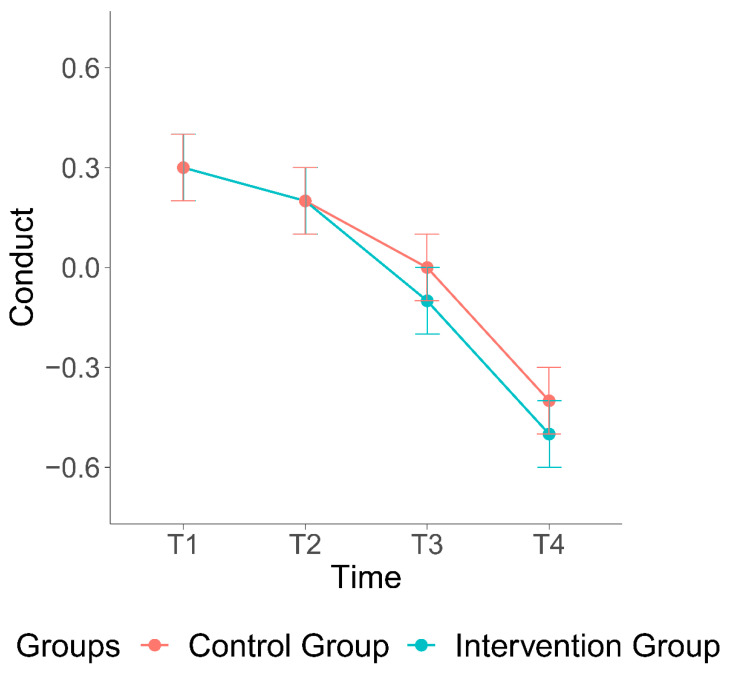
Effect of the VIPP-Intervention on conduct problems.

The first hypothesis was that children in the intervention group would exhibit a (stronger) decrease in conduct problems compared to children in the control group. The interaction between condition and squared time was significant, suggesting that the intervention reduced child conduct problems (b = −0.07, se = 0.03, *p* = 0.03, Figure 2).

**Figure 3:**
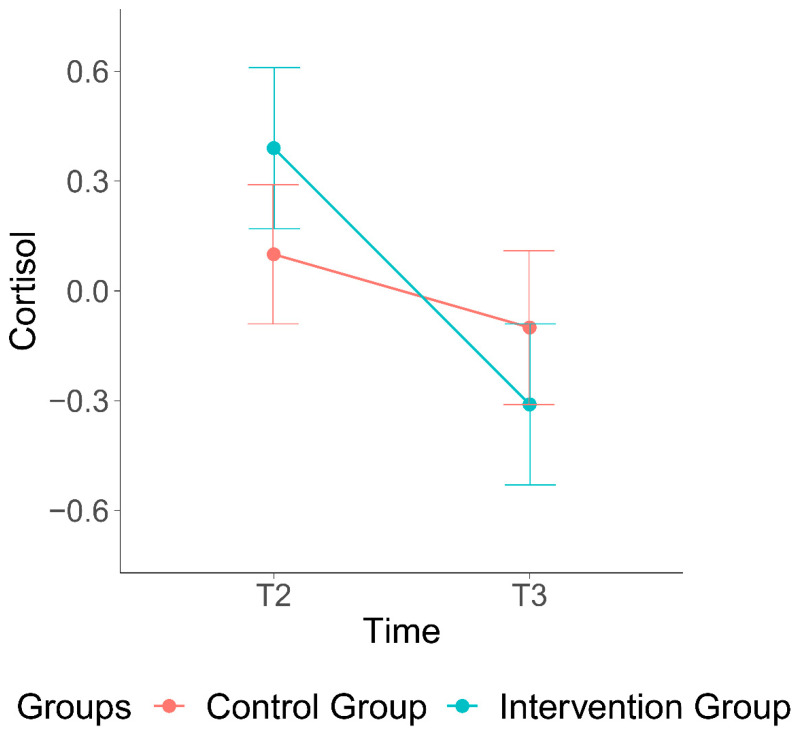
Effect of the VIPP-Intervention on hair cortisol levels.

Our second hypothesis was that the HCC of the children would be significantly affected by the intervention. The interaction between time and condition was significant, implying that in the intervention group cortisol levels decreased more compared to the control group (b = −0.44, se = 0.19, *p* = 0.02, Figure 3).

In the third hypothesis, we suggested that the intervention effects on conduct problems and HCC would be moderated by the genetic differential susceptibility of the child as marked by the PGS-ES and the PGS-SESA. For neither PGS did we find a significant three-way interaction (time^2^ × condition × PGS or time × condition × PGS, for conduct problems and HCC, respectively), suggesting that these PGSs did not moderate intervention effects on conduct problems (see Table 3) or HCC (see Table 4).

### 3.3. Sensitivity Analyses

Our sensitivity analyses revealed the following: (1) When repeating the analyses including only those participants who received all five intervention sessions (82% of the families), we found the same results of the VIPP−SD on conduct problems (b = −0.10, se = 0.04, *p* < 0.01) and on HCC (b = −0.55, se = 0.16, *p* < =0.01) as in the main analysis (see Table S5a,b). (2) In the analysis including only participants where the mother was the primary caregiver (91% of the families), results were comparable to the main analyses, although the interaction between time and condition was statistically significant only for conduct problems (b = −0.08, se = 0.04, *p* = 0.03) and fell just short of significance for HCC (b = −0.39, se = 0.22, *p* = 0.07) when using p < 0.05 as a cut-off point (see Table S5a,b). (3) The sensitivity analysis for both cohorts separately revealed that, in the early childhood cohort, the intervention×time^2^ effect on conduct problems (b = −0.06, se = 0.05, *p* = 0.26), and HCC (b = −0.34, se = 0.25, *p* = 0.16) was not significant anymore. In middle childhood, we found the same results of the VIPP−SD on conduct problems (b = −0.09, se = 0.04, *p* = 0.03) and HCC (b = −0.71, se = 0.22, *p* < 0.01) as in the main analysis (see Table S6a,b for the estimates).

## 4. Discussion

In the current study, we investigated whether a brief interaction-focused parenting intervention, the VIPP-SD, had an effect on children’s conduct problems and hair cortisol concentrations. In addition, we examined whether the intervention effect was moderated by children’s differential susceptibility captured by two novel polygenic scores. The VIPP-SD was successful in decreasing children’s conduct problems in our population-based sample. This adds new evidence to the meta-analysis of VIPP-SD studies that reported an overall non-significant effect on child externalizing behaviors [2]. In that meta-analysis, only nine studies reported on externalizing behaviors, and the mean sample size of the nine studies was 116, with only three studies including more than 100 participants. It may thus have been underpowered for detecting the effects of the strength that we found in this study. Indeed, the most recent pre-registered and best-evidence study with 300 participants did find a significant effect of VIPP-SD on externalizing behaviors [9] with an effect size that was similar to the effect found in our study.

In the intervention group, children’s hair cortisol concentrations decreased more after the intervention compared to the control group, indicating that the VIPP-SD was successful in decreasing hair cortisol levels. Our findings are in line with the only other published intervention study that investigated child hair cortisol concentrations as an outcome of a randomized trial. Poehlmann-Tynan and colleagues [17] found that after cognition-based compassion training (CBCT) with 25 parents and their children between 4 months and 5 years, child hair cortisol levels were significantly lower than those of children in the waitlist control group. We replicated these results in a well-powered twin study, implementing a parenting intervention based on attachment theory and the social learning model of coercive cycles [1,37]. A recent meta-analysis of 19 parenting intervention studies found no significant effect on salivary cortisol [12]. The difference may be explained by the fact that salivary cortisol provides a snapshot of cortisol levels at a specific time or day, and cortisol levels are known to fluctuate substantially during and across days [13]. Hair cortisol is thought to provide a more stable picture of cortisol secretion across time, and may thus indicate chronic stress levels instead of more volatile, temporary states of stress exposure or, e.g., physical exercise. However, hair cortisol is subject to confounding factors such as hair washing frequency, which can introduce a large amount of measurement error [38]. In case of uncontrolled confounding, the verdict is still open on which cortisol measurement method is the better choice.

We tested differential susceptibility to the positive effects of the intervention using two polygenic scores. We did not find support for differential susceptibility to parenting concerning conduct problems or hair cortisol concentrations. One explanation might be that susceptibility might not be domain-general but domain-specific [18,39]. For example, Zhang et al. [40] found that individual differences in susceptibility to family–social effects were not related to individual differences in susceptibility to quality-of-care cognition-related effects, indicating that individuals who were susceptible to family–social effects were not susceptible to quality-of-care effects and vice versa. However, they did find that individual differences in susceptibility to family and child-care effects were positively correlated. Considering the similarity of family effects (care environment at home) and child care effects (care environment at child care), the significant correlation between susceptibilities does not inevitably point to a domain generality as the authors suggested but rather to a broader but specific domain (caregivers). Indeed, a recent paper by Belsky et al. [41] found that approximately 50% of the children who were highly susceptible to the effect of childcare quality on pre-academic skills were not highly susceptible to the effect of child care quantity on behavior problems and vice versa.

We speculate that the two PGSs in our study might be indicators of differential susceptibility, but specific to the outcome domains for which they were developed, and not for the specific exposure or outcome domain we focused on in this parenting intervention study. The PGS-ES is a polygenic score for differential susceptibility based on GWAS data for anxiogenic environments [22]. Keers and colleagues [22] found that the PGS-ES moderated the association between child-reported parenting at age 12 and children’s internalizing emotional problems. Lemery-Chalfant et al. [23] found that the PGS-ES moderated the association between the effects of the Family Check-Up intervention and internalizing psychopathology. In the current study, we considered the possibility that susceptibility towards the effects of parenting on internalizing and externalizing behaviors might be indicated by one underlying PGS, but we were not able to corroborate this assumption. Further research is needed to investigate whether this PGS-ES is a susceptibility marker for any outcome outside the internalizing domain.

We were the first to explore the PGS-SESA, a PGS based on the GWAS of susceptibility to the environmental stress and adversity (SESA) cluster of neuroticism [24], as a moderator of the effects of a parenting intervention on children’s conduct problems and hair cortisol concentrations. Considering the possibility of domain-specificity of differential susceptibility, we can only cautiously conclude that in our study the PGS-SESA did not emerge as a differential susceptibility marker. Susceptibility to environmental stress and adversity in the context of neuroticism might be more closely related to the perception of stress and adversity than to the neurobiological experience of stress that is central to the Boyce and Ellis model of sensitivity to stress [18,39]. Another issue might be the age difference between the sample providing the PGS-SESA and the children in our study. Although DNA does not change across age, its interplay with environmental influences might fluctuate across time [42,43]. More research on the PGS-SESA is clearly required to draw firm conclusions about its role as a marker of differential susceptibility to various environments, in various ages, and for different outcomes.

Some limitations should be noted. We measured child conduct problems using parent-report questionnaires. We cannot exclude the possibility that the intervention increased parents’ appreciation or tolerance of child behaviors. Parents might have reported fewer conduct problems after the intervention because of a change in the way they interpreted their child’s behavior. However, we included the reports of both the primary parent who participated in the intervention and the other parent who was only invited to one booster session. It should also be noted that the intervention was successful in improving parental sensitive discipline in the early childhood cohort [27], whereas in the middle childhood cohort parents’ attitudes on sensitive parenting improved, but not their behavior one month after the intervention [28]. As reported in Runze et al [28], the task within which sensitive discipline was measured in the middle childhood cohort might have been too complex. The intervention may have had an effect on a dimension of parenting behavior that was not measured at post-test but was relevant for its influence on child behavior problems. As imputation strategy, we used single imputation with the Expectation Maximization, where it is not possible to include interactions as predictors. Therefore, there may be bias in the standard errors and the estimation of the interactions. Another limitation concerns the skewed distribution of conduct problem scores in our low-risk sample, decreasing the power to detect (moderated) differences between the intervention and control groups. A better distribution of the outcome measure or a much larger sample size would increase power and would also help to facilitate model convergence. Gene × Environment (G × E) interactions have been criticized because of a basic lack of statistical power similar to the test of any interaction term outside the realm of genetics but this critique focused on correlational designs [44]. In experimental designs, the statistical power of tests for (G × E) interactions is much larger [20].

Having said that, we believe that our study provides a sufficiently firm basis for the conclusions drawn. We collected data in a relatively large sample and included four waves of data collection for conduct problems. Together with the preregistration and the RCT design, this gives us confidence in the replicability of our study results. Bearing in mind that most phenotypes are influenced by many genetic variants [45], we used two novel polygenic scores to test for differential susceptibility as opposed to single candidate genes as has been done in the past. The current study is among the first set of randomized trials to use some GWAS-based polygenic scores as markers for differential susceptibility. Although we could not confirm their role as moderators of intervention effects, this approach is an important first step to the identification of the possible genetic origins of differential susceptibility in a domain with rather complex exposures as well as outcomes.

## 5. Conclusions

In this randomized controlled trial with 445 families from two twin cohorts including 890 children, we found that the Video Feedback Intervention to Promote Positive Parenting and Sensitive Discipline (VIPP-SD) was successful in decreasing conduct problems and hair cortisol concentrations in school-aged children. Intervention effects were not moderated by the two polygenic scores specifically developed for other domains and other ages, suggesting the potential age- and domain-specificity of differential susceptibility.

## Figures and Tables

**Table 1:** Characteristics of the complete sample, and separately for the intervention and control groups.

	Early Childhood Cohort			Middle Childhood Cohort		
	Total (*n* = 202)	Intervention Group (*n* = 83)	Control Group (*n* = 119)	Total (*n* = 243)	Intervention Group (*n* = 91)	Control Group (*n* = 152)
**Twin characteristics**						
Age M (SD)	3.76 (0.57)	3.74 (0.62)	3.77 (0.53)	7.92 (0.66)	7.94 (0.66)	7.92 (0.67)
Sex (% boys)	45	45.8	44.5	48.6	49.5	48.0
Country of birth (% the Netherlands)	99.5	100	99.2	99.2	100	98.7
Zygosity (% MZ)	60.4	66.3	56.3	55.1	50.5	57.9
**Family characteristics**						
Primary parent (%)						
Biological mother	91.6	88.0	94.1	90.5	87.9	92.1
Adoptive mother	0	0	0	0.8	1.1	0.7
Biological father	8.4	12.0	5.9	8.6	11.0	7.2
Age primary parent M (SD)	36.87 (4.69)	36.89 (4.82)	36.82 (4.62)	40.48 (4.66)	40.77 (4.78)	40.32 (4.60)
Country of birth (% the Netherlands)	96.0	98.8	94.1	97.5	96.7	98.0
Educational level primary parent						
Lower and Intermediate vocational	30.2	37.3	25.2	34.3	35.2	33.8
Higher vocational, university bachelor	42.1	36.1	46.2	41.7	39.6	43.0
Post-higher vocational, university master	27.7	26.5	28.6	24.0	25.3	23.2
Primary parents’ marital status (%)						
Two-parent household	96.5	96.4	96.6	93.8	93.4	94.1
Single parent household	3.5	3.6	3.4	6.2	6.6	5.9

Note. No differences between intervention and control group, if not otherwise reported; measures were taken at T1.

**Table 2:** Means And Standard deviation of the outcome variables.

	T1	T2	T3	T4
	M (*SD*)	M (*SD*)	M (*SD*)	M (*SD*)
Hair cortisol (HCC) in pg/mg				
Intervention group	na	3.25 (4.09)	3.04 (4.60)	na
Control group	na	3.08 (3.51)	3.04 (4.47)	na
Conduct problems				
Intervention group	1.30 (0.27)	1.29 (0.27)	1.26 (0.26)	1.21 (0.23)
Control group	1.30 (0.28)	1.29 (0.29)	1.26 (0.26)	1.23 (0.26)

Note. HCC was only collected at T2 and T3.

**Table 3:** Multilevel model statistics testing the intervention effect and moderator effect on conduct problems.

Predictor	Est	SE	*p*	95% CIs
Intercept	0.05	0.07	0.74	−0.06–0.16
Time	0.09	0.10	0.33	−0.06–0.25
**Time^2^**	**−0.24**	**0.10**	**0.01**	**−0.38–−0.08**
Condition	0.02	0.06	0.27	−0.08–0.11
**Condition × Time^2^**	**−0.08**	**0.04**	**0.03**	**−0.13–−0.02**
Condition × PGS-ES × Time^2^	−0.00	0.00	0.41	−0.11–0.04
Condition × PGS-SESA × Time^2^	−0.05	0.04	0.20	−0.09–0.01

Note. Condition modeled as between level; Significant estimates (*p* < 0.05) are shown in bold; standardized regression coefficients are reported; R^2^ = 2.0%.

**Table 4:** Multilevel model statistics testing the intervention effect and moderator effect on HCC.

Predictor	Est	SE	*p*	95% CIs
Intercept	0.09	0.09	0.32	−0.06–0.23
**Time**	**−0.12**	**0.05**	**0.02**	**−0.20–−0.04**
**Condition**	**0.50**	**0.19**	**0.01**	**0.20–0.81**
**Condition × Time**	**−0.44**	**0.19**	**0.02**	**−0.75–−0.13**
Condition × PGS-ES × Time	0.06	0.06	0.34	−0.04–0.15
Condition × PGS-SESA × Time	0.00	0.06	0.95	−0.09–0.10

Note. Condition modeled as between level; Significant estimates (*p* < 0.05) are shown in bold; standardized regression coefficients are reported; R^2^ = 20.5%.

## Data Availability

The data presented in this study are available on request from the corresponding author. The data will be made publicly available after October 2023.

## Supplementary Materials

The following supporting information can be downloaded at: https://www.mdpi.com/article/10.3390/ijerph192215026/s1, Table S1: Reliability of the conduct problems subscale and correlation between primary parent report and other parent report; Table S2a: R2 of the PGS-SESA at different thresholds; Table S2b: R2 of the PGS-ES at different thresholds; Table S3: Number of outliers and percentage missingness in the outcome variables; Table S4a: Frequency and Descriptives of the possible control variables; Table S4b: Testing of possible continuous covariates using regression analysis; Table S4c: Testing of possible categorical covariates of cortisol using an ANOVA; Table S5a: Multilevel model statistics testing the intervention effect and moderator effect on HCC; Table S5b: Multilevel model statistics testing the intervention effect and moderator effect on conduct problems; Table S6a: Multilevel model statistics testing the intervention effect and moderator effect on HCC in the cohorts separately; Table S6b: Multilevel model statistics testing the intervention effect and moderator effect on conduct problems in the cohorts separately; Section S2: Deviations of pre-registered analysis plan.

## Appendix A

**Table A1:** CONSORT 2010 checklist of information to include when reporting a randomized trial.

Section/Topic	Item No	Checklist Item	As Reported on Page No
Title and abstract			
	1a	Identification as a randomized trial in the title	1
	1b	Structured summary of trial design, methods, results, and conclusions (for specific guidance see CONSORT for abstracts)	1
Introduction			
Background and objectives	2a	Scientific background and explanation of the rationale	1–3
	2b	Specific objectives or hypotheses	3
Methods			
Trial design	3a	Description of trial design (such as parallel, factorial) including allocation ratio	3–5
	3b	Important changes to methods after trial commencement (such as eligibility criteria), with reasons	7, Supplement
Participants	4a	Eligibility criteria for participants	3–5
	4b	Settings and locations where the data were collected	3–5
Interventions	5	The interventions for each group with sufficient details to allow replication, including how and when they were administered	5
Outcomes	6a	Completely defined pre-specified primary and secondary outcome measures, including how and when they were assessed	4–6
	6b	Any changes to trial outcomes after the trial commenced, with reasons	7, Supplement
Sample size	7a	How sample size was determined	3
	7b	When applicable, explanation of any interim analyses and stopping guidelines	Not applicable
Randomization:			
Sequence generation	8a	Method used to generate the random allocation sequence	5
	8b	Type of randomization; details of any restriction (such as blocking and block size)	5
Allocation concealment mechanism	9	Mechanism used to implement the random allocation sequence (such as sequentially numbered containers), describing any steps taken to conceal the sequence until interventions were assigned	5
Implementation	10	Who generated the random allocation sequence, who enrolled participants, and who assigned participants to interventions	5
Blinding	11a	If done, who was blinded after assignment to interventions (for example, participants, care providers, those assessing outcomes) and how	5
	11b	If relevant, description of the similarity of interventions	Not applicable
Statistical methods	12a	Statistical methods used to compare groups for primary and secondary outcomes	7
	12b	Methods for additional analyses, such as subgroup analyses and adjusted analyses	7
Results			
Participant flow (a diagram is strongly recommended)	13a	For each group, the numbers of participants who were randomly assigned, received intended treatment, and were analyzed for the primary outcome	4
	13b	For each group, losses and exclusions after randomization, together with reasons	4
Recruitment	14a	Dates defining the periods of recruitment and follow-up	4
	14b	Why the trial ended or was stopped	Not applicable
Baseline data	15	A table showing baseline demographic and clinical characteristics for each group	4
Numbers analyzed	16	For each group, number of participants (denominator) included in each analysis and whether the analysis was by original assigned groups	4,7
Outcomes and estimation	17a	For each primary and secondary outcome, results for each group, and the estimated effect size and its precision (such as 95% confidence interval)	8–10
	17b	For binary outcomes, presentation of both absolute and relative effect sizes is recommended	Not applicable
Ancillary analyses	18	Results of any other analyses performed, including subgroup analyses and adjusted analyses, distinguishing pre-specified from exploratory	10–11, Supplement
Harms	19	All important harms or unintended effects in each group (for specific guidance see CONSORT for harms)	Not applicable
Discussion			
Limitations	20	Trial limitations, addressing sources of potential bias, imprecision, and, if relevant, the multiplicity of analyses	12
Generalizability	21	Generalizability (external validity, applicability) of the trial findings	11,12
Interpretation	22	Interpretation consistent with results, balancing benefits and harms, and considering other relevant evidence	10–12
Other information			
Registration	23	Registration number and name of trial registry	5
Protocol	24	Where the full trial protocol can be accessed, if available	Not applicable
Funding	25	Sources of funding and other support (such as supply of drugs), role of funders	13
