# Supplementary material for: Conduct Problems and Hair Cortisol Concentrations Decrease in School-Aged Children after VIPP-SD: A Randomized Controlled Trial in Two Twin Cohorts
Source: Int J Environ Res Public Health. 2022 Nov 15;19(22):15026. doi: 10.3390/ijerph192215026 (PMC9690337; doi:10.3390/ijerph192215026)
Supplement: Supplementary file 1 [file ijerph-19-15026-s001.zip › ijerph-1973423-supplementary.pdf]

Supplement Section S1

Table S1.

Reliability of the conduct problems subscale and correlation between primary parent report and other parent report

	Full Sample		Early childhood cohort		Middle childhood cohort	
	Cronbach's α	r PP×OP	Cronbach's α	r PP×OP	Cronbach's α	r PP×OP
T1	0.80	0.53	0.66	0.49	0.72	0.48
T2	0.70	0.51	0.65	0.42	0.73	0.55
T3	0.71	0.48	0.71	0.47	0.68	0.51
T4	0.74	0.55	0.82	0.52	0.74	0.54

Note. T1-T4 are the time points of measurement; Standardized Cronbach's alpha is reported; Pearson's correlations are reported, all correlations are significant at the 0.01 level; PP = primary parent; OP = other parent

Table S2a

R^2 of the PGS-SESA at different thresholds

	0.001	0.05	0.1	0.2	0.3	0.4	0.5	1
Conduct T1	**0.060**	0.045	0.029	0.021	0.020	0.013	0.006	0.007
Conduct T2	**0.079**	0.026	0.012	0.008	0.004	0.006	0.010	0.004
Conduct T3	0.026	**0.027**	0.022	0.020	0.024	0.007	0.009	0.009
Conduct T4	**0.046**	0.021	0.030	0.028	0.025	0.015	0.008	0.005
HCC T2	0.036	0.034	0.019	**0.039**	0.021	0.032	0.027	0.025
HCC T3	0.081	**0.179**	0.172	0.126	0.129	0.114	0.116	0.117

Note. HCC = Hair cortisol concentrations; Conduct = Conduct problems.

Table S2b

R^2 of the PGS-ES at different thresholds

	0.001	0.05	0.1	0.2	0.3	0.4	0.5	1
Conduct T1	0.036	0.014	0.042	0.006	0.020	0.033	0.047	**0.064**
Conduct T2	0.033	0.044	0.018	**0.064**	0.039	0.021	0.022	0.011
Conduct T3	0.013	0.003	0.013	**0.035**	0.019	0.005	0.003	0.010
Conduct T4	**0.094**	0.055	0.039	0.071	0.052	0.020	0.018	0.009
HCC T2	0.029	**0.059**	0.043	0.001	0.021	0.056	**0.059**	0.056
HCC T3	0.013	0.001	0.051	0.121	0.097	0.110	**0.132**	0.131

Note. HCC = Hair cortisol concentrations; Conduct = Conduct problems.

Table S3

Number of outliers and percentage of missingness in the outcome variables

	**T1**	**T2**	**T3**	**T4**
**Outliers**				
Hair cortisol	not assessed	7	12	not assessed
Conduct problems	4	5	4	7
**% Missing**				
Hair cortisol	not assessed	11.5	17.6	not assessed
Conduct problems	4.4	2.2	12.5	15.2

Note. T1-T4 are the time points of measurement

Table S4a.

Frequency and Descriptives of the possible control variables

	Full Sample			Early childhood cohort			Middle childhood cohort		
	Total (n = 445)	Intervention group (n = 174)	Control group (n = 271)	Total (n = 202)	Intervention group (n = 83)	Control group (n = 119)	Total (n = 243)	Intervention group (n = 91)	Control group (n = 152)
BMI M (SD)									
Wave 2	16.0 (1.7)	16.1 (1.6)	16.0 (1.7)	15.7 (1.3)	15.7 (1.3)	15.7 (1.3)	16.3 (1.8)	16.3 (1.8)	16.3 (1.9)
Wave 3	16.0 (1.9)	16.0 (1.9)	16.0 (1.9)	15.4 (1.4)	15.3 (1.3)	15.4 (1.5)	16.5 (2.1)	16.7 (2.1)	16.5 (2.1)
Last time hair wash in % [1]									
0 - 24 hours	32.0	33.3	31.0	29.2	30.0	28.6	39.8	42.0	38.1
24 - 48 hours	33.1	34.5	32.1	38.2	40.8	36.5	18.6	18.0	19.0
> 48 hours	34.9	32.2	36.9	32.6	29.2	34.9	41.6	40.0	42.9
Hair colour[1]									
Red	3.2	3.3	3.1	4.3	4.6	4.2	0	0	0
Blond	69.4	70.6	68.6	68.1	67.7	68.2	73.5	78.0	69.8
Brown	26.4	26.1	26.7	26.4	27.7	25.5	26.5	22.0	30.2
Black	0.9	0	1.6	1.2	0	2.1	0	0	0
Frequency hair wash in %[1]									
< 1 time per week	5.7	8.9	3.5	7.8	12.3	4.7	not assessed	not assessed	not assessed
1-2 times per week	60.9	57.7	63.1	63.9	61.5	65.6	52.2	48.0	55.6
3-4 times per week	26.7	26.7	26.7	22.4	21.6	22.9	39.0	40.0	38.1
> 4 times per week	6.7	6.7	6.7	5.9	4.6	6.8	8.8	12.0	6.3
Number of persons in household[2]	4.9 (0.9)	4.9 (0.8)	4.8 (0.9)	4.8 (0.8)	4.8 (0.9)	4.8 (0.8)	4.9 (1.0)	5.0 (0.8)	4.9 (1.0)

Note. [1]Assessed in T4 of the middle childhood cohort and in T6 of the early childhood cohort, [2] Assessed in T1 in the early childhood cohort and in T2 in the middle childhood cohort; sample sizes differ per variable

Table S4b.

Testing of possible continuous covariates using regression analyses

	b	se	β	t	p	R^2
(Intercept)	5.12	0.76		6.77	< .001	11%
**BMI**	**-0.11**	**0.04**	**-0.07**	**-2.60**	**.009**	
Nr of Persons in Household	0.04	0.08	0.01	0.42	.673	
**Age**	**-0.09**	**0.03**	**-0.07**	**-2.85**	**.005**	

Note. Significant values (p < .05) in bold

Table S4c.

Testing of possible categorical covariates of cortisol using an ANOVA

	**Cortisol**		
	**F**	**df**	**η**
(Intercept)	28.86		
**Cohort**	**6.55**	**1**	**0.01**
SES	0.29	2	0.00
Ethnicity	0.56	3	0.00
**Sex**	**8.60**	**1**	**0.01**
**Hair: frequency of washing**	**2.74**	**3**	**0.01**
**Hair: last wash**	**3.89**	**2**	**0.01**
Hair: colour	2.16	3	0.01

Note. Significant values (p < .05) in bold

Table S5a

Multilevel model statistics testing the intervention effect and moderator effect on HCC

	Complete treatment				Mothers only			
Predictor	Est	SE	*p*	95% CIs	Est	SE	*p*	95% CIs
Intercept	0.02	0.10	.87	-0.15 – 0.19	0.10	0.10	.30	-0.06 - 0.26
Time	**-0.11**	**0.05**	**.04**	**-0.20 – -0.02**	-0.10	0.05	.05	-0.19 - -0.02
Cnd	**0.60**	**0.17**	**<.001**	**0.33 – 0.88**	0.43	0.22	.05	0.08 - 0.79
Cnd×Time	**-0.55**	**0.16**	**<.01**	**-0.82 – -0.28**	-0.39	0.22	.07	-0.75 - -0.04
Cnd×PGS-ES×Time	0.08	0.06	.24	-0.03 – 0.18	0.04	0.07	.50	-0.06 - 0.15
Cnd×PGS-SESA×Time	0.00	0.06	.97	-0.10 – 0.10	-0.01	0.06	.89	-0.11 - 0.10

Note. Significant estimates ($p < .05$) are shown in bold, standardized regression coefficients are reported; R^2 for complete treatment = 30.5%, for complete data = 11.8%; and for mothers only = 15.7%;

Table S5b

Multilevel model statistics testing the intervention effect and moderator effect on conduct problems

	Complete treatment				Mothers only			
Predictor	Est	SE	*p*	95% CIs	Est	SE	*p*	95% CIs
Intercept	0.00	0.07	.99	-0.12 – 0.12	0.05	0.07	.48	-0.07 - 0.17
Time	-0.08	0.11	.47	-0.25 - 0.10	0.06	0.10	.55	-0.11 - 0.23
Time²	-0.08	0.10	.41	-0.25 - 0.08	**-0.21**	**0.10**	**.04**	**-0.37 - -0.04**
Condition (Cnd)	0.01	0.06	.91	-0.10 - 0.11	0.01	0.06	.84	-0.09 - 0.11
Cnd×Time	**-0.10**	**0.04**	**<.01**	**-0.16 - -0.04**	**-0.08**	**0.04**	**.03**	**-0.14 - -0.02**
Cnd×PGS-ES×Time	-0.00	0.05	.98	-0.09 – 0.09	-0.03	0.05	.51	-0.11 - 0.05
Cnd×PGS-SESA×Time	-0.06	0.04	.10	-0.12 – 0.00	-0.03	0.03	.29	-0.09 - 0.02

Note. Significant estimates ($p < .05$) are shown in bold, standardized regression coefficients are reported; R^2 for complete treatment = 2.8%, for complete data = 6.6%, and for mothers only = 2.2%;

Table S6a

Multilevel model statistics testing the intervention effect and moderator effect on HCC in the cohorts separately

	Early Childhood				Middle Childhood			
Predictor	Est	SE	*p*	95% CIs	Est	SE	*p*	95% CIs
Intercept	0.13	0.10	.18	-0.03 - 0.30	-0.07	0.22	.75	-0.42 - 0.28
Time	**-0.13**	**0.06**	**.03**	**-0.23 - -0.03**	-0.06	0.06	.34	-0.17 - 0.04
Condition (Cnd)	0.41	0.24	.09	0.01 - 0.80	**0.80**	**0.17**	**<.001**	**0.51 - 1.08**
Cnd×Time	-0.34	0.25	.16	-0.75 - 0.06	**-0.71**	**0.22**	**<.01**	**-1.06 - -0.35**
Cnd×PGS-ES×Time	-0.01	0.07	.87	-0.13 - 0.11	0.19	0.10	.07	0.02 - 0.36
Cnd×PGS-SESA×Time	0.04	0.07	.57	-0.08 - 0.15	-0.08	0.08	.30	-0.21 - 0.05

Note. Significant estimates (p < .05) are shown in bold, standardized regression coefficients are reported; R^2 for the early childhood cohort = 14.9% and for the middle childhood cohort = 45.4%;

Table S6b

Multilevel model statistics testing the intervention effect and moderator effect on conduct problems in the cohorts separately

	Early Childhood				Middle Childhood			
Predictor	Est	SE	*p*	95% CIs	Est	SE	*p*	95% CIs
Intercept	**0.24**	**0.09**	**<.01**	**0.09 - 0.39**	**-0.22**	**0.10**	**.04**	**-0.39 - -0.05**
Time	0.23	0.14	.11	-0.01 - 0.46	-0.03	0.13	.81	-0.25 - 0.19
$Time^2$	**-0.35**	**0.13**	**<.01**	**-0.57 - -0.13**	-0.12	0.13	.38	-0.33 - 0.10
Cnd	-0.06	0.08	.48	-0.20 - 0.08	0.06	0.08	.45	-0.08 - 0.20
Cnd×$Time^2$	-0.06	0.05	.26	-0.14 - 0.03	**-0.09**	**0.04**	**.03**	**-0.16 - -0.02**
Cnd×PGS-ES×$Time^2$	-0.00	0.06	.95	-0.11 - 0.10	0.03	0.07	.68	-0.08 - 0.14
Cnd×PGS-SESA×$Time^2$	-0.06	0.04	.16	-0.14 - 0.01	-0.02	0.04	.66	-0.09 - 0.05

Note. Significant estimates (p < .05) are shown in bold, standardized regression coefficients are reported; R^2 for the early childhood cohort = 1.9% and for the middle childhood cohort = 2.5%;

Section S2

Deviations of pre-registered analysis plan

We deviated from our pre-registered analysis plan with regards to three aspects.

1. Mediation model

Hypotheses and/or Estimates

[…] Moreover, we expect that HCC mediates the effect of the intervention on conduct problems (H4) (see Figure 1).

Figure 1

Hypotheses depicted in a path model

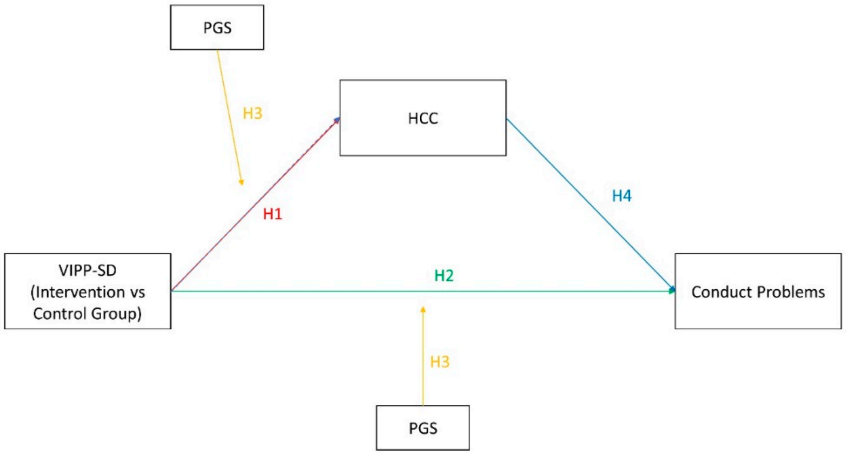

Note. Multilevel structure of the data omitted for readability; Colors indicate the different hypotheses that will be tested within this model; HCC = hair cortisol concentration; PGS = polygenic score of differential susceptibility; VIPP-SD = video feedback intervention to promote positive parenting and sensitive discipline

Unfortunately, it was not possible to test a mediation model entirely. We measured hair cortisol concentrations twice (1 pre- and 1 post-measurement). This required us to include time as a linear predictor, as well as the interaction terms time×condition, time×condition×PGS-ES and time×condition×PGS-SESA. At the same time, we measured conduct problems 4 times (2 pre- and 2 post-measurements) requiring us to include a quadratic time predictor. That means including time²,time²×condition, time²×condition×PGS-ES and time²×condition×PGS-SESA. Moreover, there were different p-value thresholds for the PGSs with regard to conduct problems or hair cortisol concentration, which means that we would have had to include both PGSs twice with differing p-value thresholds. However, the correlations between time and time² and the different threshold PGSs was too high, therefore

the model could not converge. When including only 1 of the several measures, results are not interpretable. Therefore, we decided to drop the last hypothesis and focus on the individual effects of the VIPP-SD on conduct problems and hair cortisol.

2. Exploratory analysis

Exploratory analysis

[…] Furthermore, we will exploratory run the analyses with the subscale prosocial behavior as outcome to investigate whether the VIPP-SD also has an effect on positive behaviors, such as prosocial behavior.

We exploratory assessed the effect of the VIPP-SD on prosocial behavior, but we chose to not report it in the main manuscript because we found it to be irrelevant for the main message about conduct problems and hair cortisol concentrations. However, we still report the results on prosocial behavior in the supplementary materials and are completely open about our exploratory analysis (see Table S7, S8a,b, S9, S10 and S11).

Table S7

Reliability of the prosocial behavior subscale and correlation between primary parent report and other parent report

	**Full Sample**		**Early childhood cohort**		**Middle childhood cohort**	
	Cronbach's α	r PP*OP	Cronbach's α	r PP*OP	Cronbach's α	r PP*OP
T1	0.87	0.47	0.79	0.44	0.61	0.47
T2	0.76	0.48	0.76	0.48	0.77	0.46
T3	0.74	0.43	0.75	0.39	0.72	0.47
T4	0.83	0.40	0.85	0.60	0.79	0.51

Note. T1-T4 are the time points of measurement; Standardized Cronbach's alpha is reported; Pearson's correlations are reported, all correlations are significant at the 0.01 level; PP = primary parent; OP = other parent

Table S8a

R^2 of the PGS-SESA at different thresholds

	0.001	0.05	0.1	0.2	0.3	0.4	0.5	1
Prosocial T1	0.016	0.002	0.005	0.008	0.003	0.021	**0.025**	0.023
Prosocial T2	0.009	**0.056**	0.054	0.054	0.035	0.054	0.055	0.051
Prosocial T3	0.002	**0.060**	0.036	0.027	0.016	0.037	0.039	0.035
Prosocial T4	0.015	0.021	0.047	0.048	0.043	0.063	0.068	**0.071**

Table S8b

R^2 of the PGS-ES at different thresholds

	0.001	0.05	0.1	0.2	0.3	0.4	0.5	1
Prosocial T1	0.031	0.008	**0.037**	0.030	0.013	0.025	0.019	0.022
Prosocial T2	0.014	0.010	0.003	**0.050**	0.041	0.047	**0.050**	0.040
Prosocial T3	0.006	0.026	0.013	0.024	0.036	0.052	**0.054**	0.047
Prosocial T4	0.037	0.045	0.038	0.029	**0.071**	0.063	0.068	0.068

Table S9

Number of outliers and percentage missingness in the outcome variables

	**T1**	**T2**	**T3**	**T4**
**Outliers**				
Prosocial behavior	5	5	5	1
**% Missing**				
Prosocial behavior	4.4	2.2	12.5	15.2

Table S10

Multilevel model statistics testing the intervention effect and moderator effect on Prosocial behavior

Predictor	Est	SE	*p*	95% CIs
Intercept	-0-06	0.03	.04	-0.60 - -0.07
$Time^2$	**-0.03**	**0.01**	**<.001**	**-0.14 - 0.08**
Condition (Cnd)	-0.27	.93	.77	-0.39 - 0.27
Cnd×$Time^2$	0.01	0.01	.49	-0.04 - 0.11
Cnd×PGS-ES×$Time^2$	0.01	0.00	.09	0.01 - 0.50
Cnd×PGS-SESA×$Time^2$	0.00	0.02	.99	-0.09 - 0.09

Note. Significant estimates ($p < .05$) are shown in bold, standardized regression coefficients are reported; R^2 = 12.1%

3. Number of time points

There are […] three [data points] for conduct problems (repeated measures)

At the moment of the actual data analysis, we had data of four time points instead of three. Also, in the mediation model, we wanted to use conduct problems assessed after cortisol. Because we did not test the mediation model anymore, we added the data on conduct problems of T3. As can be seen in Figure 1 in the main manuscript, results would not have been different for the effect of the VIPP-SD on conduct problems.

4. Power analysis

In the main article, we report the a priori power analysis and a short summary of the posteriori power analysis, but here we additionally explain our posthoc power analyses: We computed post-hoc power using the software MLPowSim (Browne & Golalizadeh, 2009) to create an R script using the lme4 package (Bates et al., 2015). We ran 100 simulation studies with our data for both HCC and conduct problems separately. Power was excellent (>.99) to detect the VIPP-SD effect on HCC, but poor to detect the VIPP-SD effect on conduct problems (.27) introducing the risk of a false positive result (Button et al., 2013). There was excellent power to detect a three-way interaction between condition, $time^2$ and the PGS-ES on conduct problems (>.99), but for all other estimates power was insufficient (see Table S8).

Table S11

Power simulation study using MLPowerSIm/R

Predictor	Estimate	Cortisol	Conduct
	Lower bound	0.341	0.146
Intercept	Estimate	0.345	0.146
	Upper bound	0.348	0.147
	Lower bound	0.278	0.754
Time(^{2})	Estimate	0.281	0.759
	Upper bound	0.284	0.764
	Lower bound	**1**	0.266
Condition× Time(^{2})	Estimate	**1**	0.268
	Upper bound	**1**	0.27
	Lower bound	0.057	0.139
Condition× Time(^{2})×PGS-SESA	Estimate	0.057	0.14
	Upper bound	0.057	0.142
	Lower bound	0.241	**1**
Condition× Time(^{2})×PGS-ES	Estimate	0.243	**1**
	Upper bound	0.245	**1**

Note. Power estimated were computed using the standard error method

R script to compute power for the effect of the VIPP-SD on conduct problems

```
###     A programme to obtain the power of parameters in 2 level
#       balanced model  with  Normal response
#                   generated on 01/09/22
###~~~~~~~~~~~~~~~~~~~   Required packages  ~~~~~~~~~~~~~~~~~~~~~~###
   library(MASS)
   library(lme4)
###~~~~~~~~~~~~~~~~~~~~~     Initial inputs    ~~~~~~~~~~~~~~~~~~~~~###

set.seed(1)
siglevel<-0.050
z1score<-abs(qnorm(siglevel))
simus<-100
n1low<-2
n1high<-2
n1step<-0
n2low<-257
n2high<-257
n2step<-0
npred<-4
randsize<-1
```

```
beta<-c(0.049000,-0.137000,-0.071000,-0.040000,-0.037000)
betasize<-length(beta)
effectbeta<-abs(beta)
sgnbeta<-sign(beta)
randcolumn<-0
meanpred<-c(0,-0.121000,0.002000,0.007000,7.461000)
varpred<-matrix(c(0.900000,-0.136000,0.066000,-2.382000,-0.136000,0.848000,-
0.448000,0.599000,0.066000,-0.448000,0.805000,-0.391000,-2.382000,0.599000,-
0.391000,153.106000),npred,npred)
varpred2<-
matrix(c(0.000000,0.000000,0.000000,0.000000,0.000000,0.000000,0.000000,0.000000,0.00
0000,0.000000,0.000000,0.000000,0.000000,0.000000,0.000000,0.000000),npred,npred)
```
